# Publication Bias in Epidemiological Studies of Malocclusions in Mexican Children and Teenagers: A Systematic Review and Meta-Analysis

**DOI:** 10.3390/children13040580

**Published:** 2026-04-21

**Authors:** Liliana Argueta-Figueroa, Karina Alejandra Quiroz-Carlín, Mario Alberto Bautista-Hernández, Rafael Torres-Rosas, María Eugenia Marcela Castro-Gutiérrez, Yobana Pérez-Cervera, Adriana Moreno-Rodríguez, Alfonso Enrique Acevedo-Mascarúa, Enrique Antonio Martínez-Martínez

**Affiliations:** 1 SECIHTI—Tecnológico Nacional de México/Instituto Tecnológico de Toluca, Metepec 52149, Estado de Mexico, Mexico; 2 Laboratorio de Medicina Complementaria, Centro de Estudios en Ciencias de la Salud y la Enfermedad, Facultad de Odontología, Universidad Autónoma “Benito Juárez” de Oaxaca (UABJO), Universidad Avenue, “Ex-Hacienda de Cinco Señores”, Oaxaca de Juarez 68120, Oaxaca, Mexico; karca215@outlook.es (K.A.Q.-C.); yperez.cat@uabjo.mx (Y.P.-C.); 3 Departamento de Ortodoncia, Centro de Estudios en Ciencias de la Salud y la Enfermedad, Facultad de Odontología, Universidad Autónoma “Benito Juárez” de Oaxaca (UABJO), Universidad Avenue, “Ex-Hacienda de Cinco Señores”, Oaxaca de Juarez 68120, Oaxaca, Mexico; 4 Programa de Doctorado en Biociencias (SNP 005846), Facultad de Medicina y Cirugía, Universidad Autónoma “Benito Juárez” de Oaxaca (UABJO), Ex Hacienda de Aguilera S/N, Sur, San Felipe del Agua 68020, Oaxaca, Mexico; 5 Facultad de Ciencias Químicas, Universidad Autónoma “Benito Juárez” de Oaxaca (UABJO), Oaxaca de Juarez 68120, Oaxaca, Mexico

**Keywords:** risk of bias, epidemiological studies, malocclusions, prevalence, dentistry, orthodontic

## Abstract

Objective: To determine the publication bias of the reported prevalence of malocclusions in Mexican children and adolescents. Background: Publication bias determination is crucial in a systematic review, helping to ensure the conclusions’ validity and reliability. Nevertheless, without accurate knowledge of disease prevalence and patterns, the health system risks inefficiency, inequity, and failure to meet the population’s needs. On the other hand, malocclusions can impair proper chewing efficiency, contributing to digestive alterations, and nutritional deficiencies among other functional, psychological, and social problems. The data of the prevalence of malocclusion is imperative to implement early interventions in health services that prevent more severe skeletal discrepancies and reduce the need for invasive treatments in adolescence or adulthood. Methods: Studies were collected from five databases, following the PRISMA and Cochrane guidelines for systematic reviews. Eligibility criteria were full-text research in which the prevalence of malocclusions was reported. The risk of bias (Hoy tool), publication bias (the Doi plot and the Luis Furuya-Kanamori (LFK) index), and quality assessments (GRADE tool) were performed. The data were combined using a random-effects meta-analysis. Results: The result of the meta-analysis suggests a high prevalence of malocclusions in mixed dentition was 50.2% (95% confidence interval [CI]: 38.9–61.5%). However, the studies showed a risk of bias and publication bias. Conclusions: In Mexico, there is a high prevalence of malocclusions among children and adolescents. However, these results are not robust enough to draw solid conclusions, due to the low certainty of the evidence.

## 1. Introduction

Malocclusions can impair proper chewing efficiency, contributing to digestive problems and nutritional deficiencies. Additionally, they can influence phonetics, making particular speech sounds difficult to articulate, particularly in severe overjet or open bite cases [1]. Beyond these functional limitations, malocclusions can have profound psychosocial effects. Studies indicate that children with noticeable dental misalignments may experience low self-esteem, social stigma, and bullying, leading to anxiety and reduced overall well-being [2].

Publication bias occurs when studies with statistically significant results are more likely to be published [3]. Publication bias determination is crucial in a systematic review, as it helps to ensure the validity and reliability of the conclusions. First, the analysis of publication bias helps to prevent overestimating effects. It is well known that studies with positive or statistically significant results are more likely to be published, while negative or inconclusive findings may remain unpublished. This can lead to an overestimation of treatment effects or associations. Second, assessing publication bias enhances the evidence quality. Identifying and addressing potential bias improves the robustness of the synthesis, making the review more representative of all available data. Third, it reduces the risk of false conclusions. If only studies with favorable results are included, a systematic review may produce misleading interpretations, potentially affecting clinical decisions and policy-making. Fourth, evaluating publication bias supports more comprehensive data inclusion. By assessing this bias, reviewers can attempt to incorporate unpublished studies, registered trials, or the gray literature, resulting in a more balanced and accurate analysis. Finally, publication bias assessment improves transparency and reproducibility, because acknowledging potential biases allows researchers and readers to interpret findings appropriately, strengthening the credibility of the review [4,5,6].

Standard methods for detecting publication bias include the Funnel plot, Egger’s test, Begg’s test, skewness, and the trim-and-fill method. Addressing publication bias helps us to maintain the integrity of systematic reviews and meta-analyses by ensuring that they provide trustworthy evidence for decision-making [7].

Disease prevalence and pattern knowledge is critical for the structure of health service systems in these countries, because it provides a data-driven foundation for designing, implementing, and optimizing healthcare policies and infrastructure. Without accurate knowledge of disease prevalence and patterns, health systems risk inefficiency, inequity, and failure to meet population needs. Leveraging this knowledge ensures that systems are resilient, cost-effective, and capable of promoting the overall well-being of a country’s population [8,9].

Economic disparities between developing and high-income countries have a direct impact on access to dental evaluation, diagnosis, and treatment. In many resource-limited settings, orthodontic care remains predominantly a private service and is often financially inaccessible, with public dental systems prioritizing urgent or operative treatments over occlusal and craniofacial conditions [10]. Developing countries typically depend on epidemiological data from First World countries [11]. However, this practice can generate bias in the public health perspective and the decision-making process, due to the dissimilarity between the economic conditions [12]. Also, there is diversity in the epidemiological data due to the variability of health problems within a nation, which is exemplified by the fact that regions across the world encounter differences in the range of diseases, risk factors, healthcare access, health equity, and geographical characteristics. However, in dentistry, the racial epidemiological disparity could be related to racial characteristics or the study’s quality in common diseases related to morphological patterns, such as malocclusions. Therefore, evaluating publication bias becomes essential for oral public health planning, as national policies and resource allocation depend heavily on published prevalence estimates.

One might think that the potential for publication bias lies in clinical trials where one or more interventions are tested. However, observational studies, which include epidemiological studies, are not exempt from this type of bias. In the particular case of malocclusion prevalence, the overestimated results may be more striking and more easily published. The dental professional community appears to show a certain predilection for results that generate greater concern and that emphasize the urgent treatment needs of the population. Finally, it is important to note that determining the extent of publication bias is difficult, and, therefore, it is important to determine the effect of unpublished data on the conclusions of a systematic review based on the synthesized evidence of published studies. The latter, to ensure robust conclusions, it is necessary not only to assess the risk of bias related to the methodological study design, but also to evaluate publication bias [13]. Due to the former, this work aims to determine the publication bias of the reported prevalence of malocclusions in Mexican children and adolescents.

## 2. Materials and Methods

### 2.1. Protocol Registry

The review was conducted according to the Preferred Reporting Items for Systematic Reviews and Meta-Analyses (PRISMA) guidelines and the Cochrane handbook [14]. In addition, the review protocol has been registered in the International Prospective Register of Systematic Reviews (PROSPERO), which is available at https://www.crd.york.ac.uk/PROSPERO/view/CRD42024560860 (access on 16 April 2026).

### 2.2. Eligibility Criteria and Participant Characteristics of Studies

The eligibility criteria were defined according to the PICO strategy (Population and Outcome), as shown in Table 1. The included studies must be observational studies that are capable of answering the research question. On the other hand, experimental studies, case reports, case series, editor letters, short communications, in vitro studies, animal studies, comments, literature reviews, and reviews were excluded.

#### 2.2.1. Population

Mexican children ≤ 15 years old. We selected this cutoff because childhood and early adolescence extend up to approximately 15 years of age, which coincides with the end of the mixed dentition period.

#### 2.2.2. Outcomes

Prevalence rates of malocclusions.

### 2.3. Search Strategy and Databases Used

The algorithms used for the search strategy are shown in Table 1. Two reviewers [KAQC and MABH] searched five electronic databases: MEDLINE/PubMed, Dentistry & Oral Sciences Source, Scopus, Google Scholar, and Web of Science in November 2023. Given that Google Scholar displays a maximum of 1000 results per query, the search period was subdivided into smaller intervals. After identifying 7900 records with our initial algorithm, we first used a 5-year interval (1980–2010) and subsequently employed 2-year intervals to systematically retrieve all records without truncation. The manual search was achieved by examining the references from the included studies in this review.

### 2.4. Study Selection

Two reviewers (KAQC and RTR) independently screened the titles and abstracts of all search results to identify potentially eligible studies. The full text of these studies was then retrieved and independently examined to confirm eligibility according to the predefined criteria. Disagreements at any step were resolved by a third reviewer (LAF).

### 2.5. Data Collection Process and Data Items

A standardized Microsoft Excel worksheet (Microsoft Corp., Redmond, WA, USA) was developed to record relevant information from all studies included in the systematic review, comprising participant demographics, the diagnostic methodology, and outcome data. The outcome data concerning the diagnosis were homogenized as normal occlusion and malocclusion and expressed as a percentage rate. Although Angle originally classified Classes I, II, and III as distinct types of malocclusions, for epidemiological purposes, Class I is often considered a “baseline” or “neutral” sagittal relationship. This is because Class I corresponds to an anatomically normal molar relationship, even when dental irregularities are present. In contrast, Classes II and III reflect true sagittal discrepancies that constitute clinically relevant pathological variants. Therefore, for the purposes of estimating the prevalence of clinically meaningful malocclusion, we grouped Class I as the non-pathological condition and Classes II and III as the pathological categories. DAI scores were dichotomized using the standard cutoff point, classifying individuals as having no malocclusion or having clinically significant malocclusion (including the categories definite malocclusion, severe malocclusion, and very severe). Two reviewers (KAQC and MABH) were responsible for data extraction.

### 2.6. Risk of Bias in Individual Studies and Quality Assessments

To assess the risk of bias of the studies included in the review, the guidelines from the Cochrane Handbook for Systematic Reviews of Interventions Chapter 8 [15] and the Hoy et al. tool [16] were used. The risk of bias assessment was performed by two reviewers (MEMCG and MABH). In accordance with previously published reviews, the signaling questions were scored separately. The overall bias score was assigned to each included study as follows: a low risk of bias was defined as ≤1 question indicating high risk, moderate risk of bias as 2–3 questions indicating high risk, and high risk of bias as >3 questions indicating high risk [17].

For the assessment of the overall quality of evidence, the GRADE (Grading of Recommendations, Assessment, Development, and Evaluation) approach was used. The confidence intervals, heterogeneity of the source studies, risk of bias, and the presence of publication bias were used as the indicators for up- or down-grading of the evidence.

### 2.7. Meta-Analyses Methodology

The meta-analysis and publication bias tests were performed using the meta package (version 4.4.3), using R (R software version 4.4.0; R Development Core Team, 2011).

Pooled prevalence estimates were calculated using a random-effects model with the inverse-variance method. The between-study variance (τ^2^) was estimated using the Sidik–Jonkman estimator [18], and the Hartung–Knapp adjustment was applied to compute 95% confidence intervals for the pooled estimate. The statistical heterogeneity was evaluated using Cochran’s Q test (with *p* < 0.05 indicating statistical significance) and quantified using the I^2^ statistic [19]. Subgroup analyses were performed according to the risk of bias (high, moderate, or low) and publication type (thesis vs journal article), sample type (clinic-based vs community based), diagnostic method (angle classification, DAI), and rigorous review (yes/no based on the high-quality index JCR or Scopus).

Publication bias was assessed using the Doi plot and the Luis Furuya-Kanamori (LFK) index, with values > 1 indicating asymmetry and values > 2 suggesting major asymmetry. The LFK index was calculated as the intercept from a linear regression of the *z*-score (effect size divided by its standard error) against precision (the reciprocal of the standard error [1/standard error]) [20,21].

## 3. Results

### 3.1. Selection and Characteristics of the Studies

**Figure 1:**
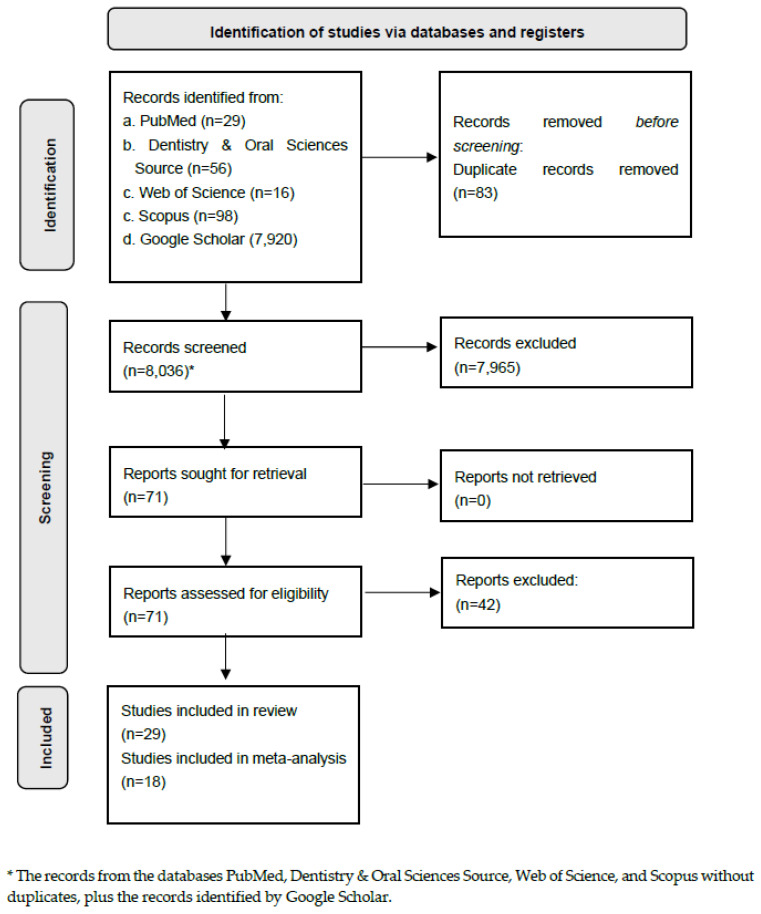
PRISMA flow diagram of the selection process in the included studies.

The initial search yielded 8164 records. After the removal of 83 duplicate records, 8036 records remained for the title and abstract screening. Of these, 71 records were identified as potentially answering the review question; however, only 29 met the eligibility criteria, as illustrated in Figure 1. Manually searching the reference lists of the included studies did not yield any further eligible records. Among the included studies, 23 reported malocclusions in mixed dentition, while six reported malocclusions in deciduous dentition. Also, in these studies, the malocclusion outcome was diagnosed based on molar relationships, using criteria such as Angle classification and the Dental Aesthetic Index (DAI), and terminal planes, for mixed and deciduous dentition, respectively, and the bite abnormalities were reported. On the other hand, the studies that did not fully meet the eligibility criteria are shown in Appendix A Table A1. Finally, the characteristics of the population and extracted data are shown in Table A2 (mixed dentition) and Table A3 (deciduous dentition).

### 3.2. Risk of Bias and Quality Assessments

**Figure 2:**
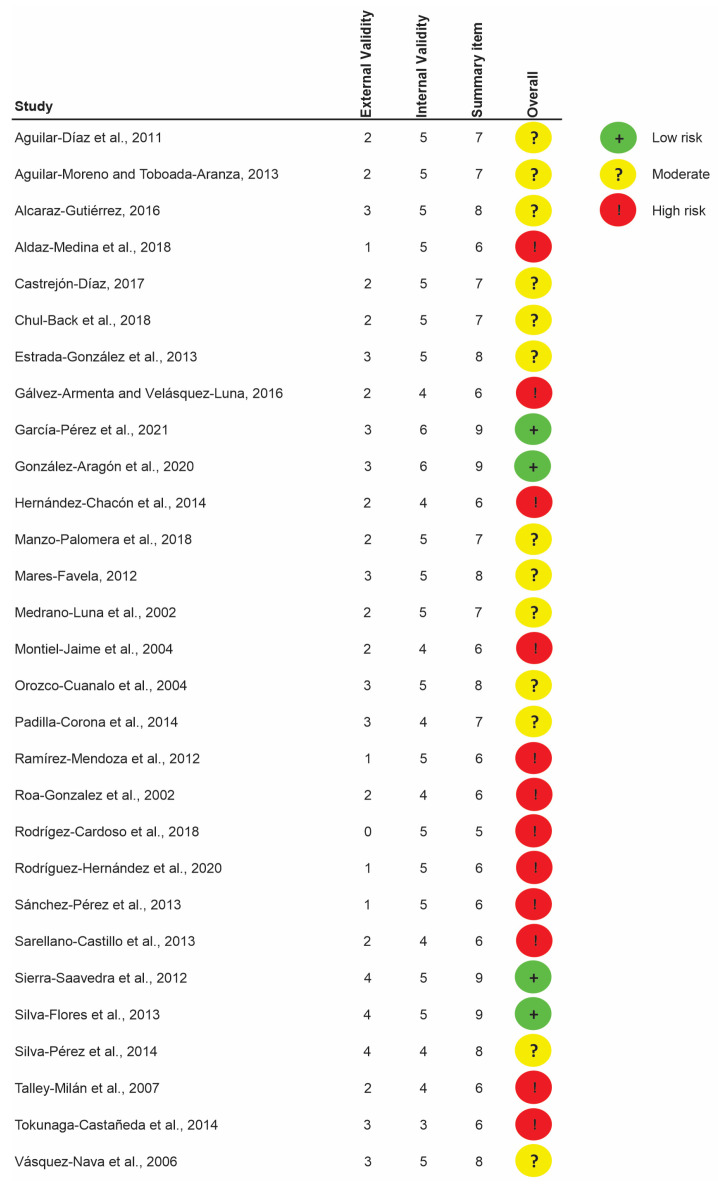
Graphical representation of the risk of bias of included studies, as assessed using the Hoy tool.

Using the risk of bias tool proposed by Hoy et al. [16], the overall risk of bias among the 36 included studies was assessed as follows: four studies (11.1%) were rated as having a low risk of bias, 15 studies (41.7%) as having a moderate risk, and 17 studies (47.2%) as having a high risk. The main concerns leading to the downgrading of studies were the use of convenience samples, such as school-based or clinic-based populations, which limits representativeness and compromises external validity. Another important limitation affecting the external validity is the lack of random selection procedures in the included studies, as illustrated in Figure 2.

### 3.3. Meta-Analyses

A meta-analysis of the prevalence rates was conducted using studies that reported malocclusion in the mixed dentition. A total of 18 studies including 5987 participants were incorporated into the quantitative synthesis; data from participants aged ≤ 15 years were extracted from two studies [22,23]. The meta-analysis of deciduous dentition was not performed due to the limited number of available studies, in accordance with the current recommendations for evidence synthesis [24].

**Figure 3:**
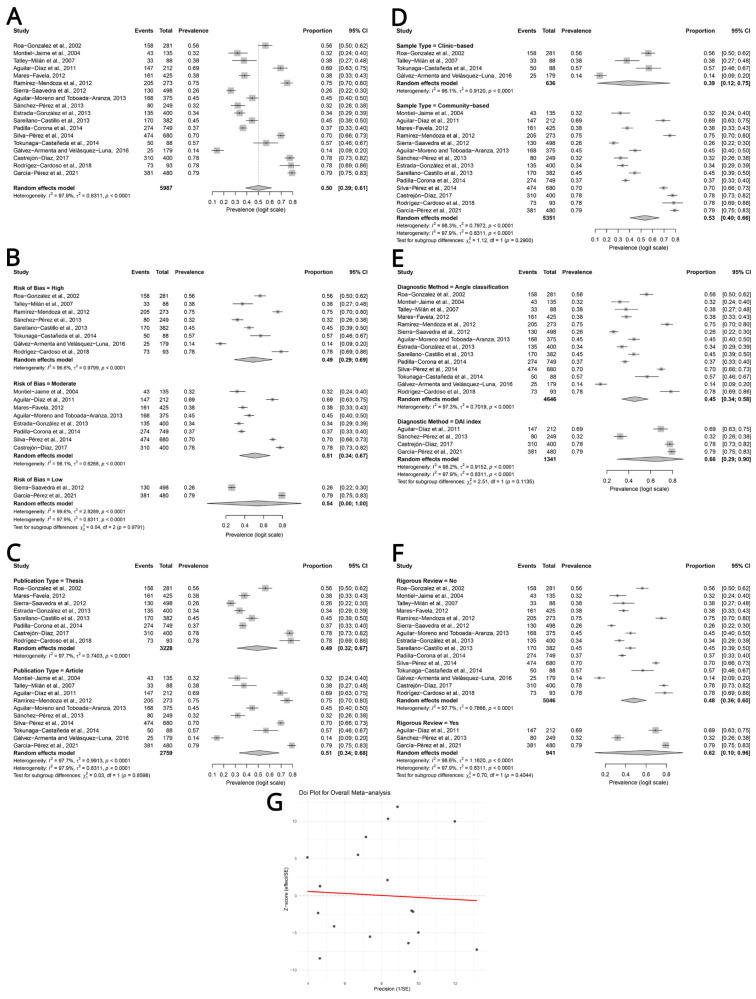
Meta-analysis of malocclusion prevalence in mixed dentition across included studies: (**A**) overall Forest plot, (**B**) Forest plot stratified by bias, (**C**) Forest plot stratified by publication type, (**D**) Forest plot stratified by sample typer, (**E**) Forest plot stratified by diagnostic method, (**F**) Forest plot stratified by rigorous review, and (**G**) Doi plot.

The overall pooled prevalence of malocclusion in mixed dentition was 50% (95% confidence interval [CI]: 39–61%), using a random-effects model with the Hartung–Knapp–Sidik–Jonkman adjustment. Substantial statistical heterogeneity was observed across studies (I^2^ = 97.9%, Cochran’s Q test *p* < 0.001), as presented in Figure 3A.

In subgroup analyses based on the risk of bias, the pooled prevalence was 49% (95% CI: 29–69%) for the eight studies with a high risk of bias, 51% (95% CI: 34–67%) for the eight studies with a moderate risk, and 54% (95% CI: 0–100%) for the two studies with a low risk of bias. No statistically significant difference in the risk of bias subgroups was identified (*p* = 0.979), see Figure 3B.

Meanwhile, when stratifying by publication type, the eight theses yielded a pooled prevalence of 49% (95% CI: 32–67%), while the ten articles gave 51.0% (95% CI: 34–68%). The difference was not statistically significant (*p* = 0.859), as shown in Figure 3C.

In subgroup analyses by sample type, the four clinic-based studies showed a pooled prevalence of 39% (95% CI: 12–75%), and the 14 community-based studies showed a pooled prevalence of 53% (95% CI: 40–66%). No statistically significant difference was observed between the two subgroups (*p* = 0.290), see Figure 3D.

Meanwhile, when stratifying by diagnostic method, the pooled prevalence was 45% (95% CI: 34–58%) for the 14 studies that use the Angle classification and 66% (95% CI: 29–90%) for the four studies using the DAI. No significant differences were found between the two subgroups (*p* = 0.113), see Figure 3E.

Simultaneously, in subgroup analyses based on rigorous review, the 15 studies without rigorous review showed a pooled prevalence of 48% (95% CI: 36–60%), whereas the 3 studies with rigorous review showed a pooled prevalence of 62% (95% CI: 10–96%). No significant difference was observed between the two subgroups (*p* = 0.404), as shown in Figure 3F. Finally, the Doi plot revealed asymmetry, with an LFK index of 1.116, indicating asymmetry, see Figure 3G.

### 3.4. Certainty of Evidence According to GRADE

Finally, the overall certainty of evidence from studies addressing mixed dentition was rated as being low regarding the true prevalence of malocclusion. The pooled estimate of 50% is based on eight studies with a high risk of bias, eight with a moderate risk of bias, and only two with a low risk of bias. The overall estimate may be influenced by limitations in sampling or outcome measurement, with substantial heterogeneity (I^2^ 97.9%, *p* < 0.001) and a wide confidence interval (<22 percentage points). The true prevalence could range from 39% to 61%. Also, publication bias is suspected, as shown in Table 2.

### 3.5. Malocclusion in Deciduous Dentition

Six studies reported on malocclusion in children with deciduous dentition, comprising a total of 2214 participants. Three studies were community-based, and three were clinic-based. Regarding risk of bias, two studies showed a high risk, three a moderate risk, and one a low risk. Three studies were published as articles and three as theses.

All studies reported the prevalence of malocclusion based on clinical examination, using either the terminal plane classification or the presence of specific bite abnormalities, such as open bite, crossbite, or overbite. The prevalence of malocclusion varied considerably across studies from Aldaz-Medina, 2018 [25], with the lowest (27%) to Hernández-Chacón et al., 2014 [26], with the highest (95.1%) frequencies of specific bite abnormalities.

With the small number of studies, the precision of any pooled estimate would be very low, and the assessment of heterogeneity and publication bias would be unreliable. Also, substantial clinical and methodological heterogeneity was shown. As a consequence, a meta-analysis of the prevalence of malocclusion in deciduous dentition was not conducted.

## 4. Discussion

Understanding the prevalence of malocclusions in a country is essential for developing effective public health policies, optimizing resource allocation, and improving overall oral health outcomes. From a healthcare planning perspective, knowing the national burden of malocclusions aids in workforce distribution and policy decisions regarding orthodontic treatment coverage in public health insurance programs. Countries with a high prevalence may need to invest in preventive and early intervention strategies to reduce the demand for complex and costly treatments in adulthood. Ultimately, prevalence studies provide a scientific foundation for evidence-based decision-making, ensuring that oral health interventions are targeted, equitable, and effective in improving population health [27,28].

According to G. Lombardo et al., the worldwide prevalence of malocclusion was 56% (95% CI: 11, 99). The highest prevalence was in Africa (81%) and Europe (72%), followed by America (53%) and Asia (48%). The malocclusion prevalence score did not change from primary to mixed dentition with a common score of 54%. Most of the included studies showed a high risk of bias (10/14 assessed by the JBI Checklist, but this is not the appropriated tool to evaluate epidemiological studies’ risk of bias) [29]. On the other hand, H. Chen et al., reported that the highest percentage of malocclusion in primary dentition was in Asia (61.81%), followed by Europe (61.50%), South America (52.69%), and Africa (32.50%). Most of the included studies showed good quality (33/47 assessed by Newcastle–Ottawa), but Newcastle–Ottawa is also not the appropriated tool to evaluate epidemiological studies’ risk of bias [30]. These results are consistent with the present review from Mexico, which estimates a prevalence of 50% in mixed dentition. However, the studies included showed a risk of bias and low certainty of evidence.

Policy- and decision-making that relies on recommendations from systematic reviews and metanalyses can be impacted by the selective publishing of research based on the nature and direction of findings. Consequently, the publication bias assessing tools are essential to ensure that decisions are based on an ethical, transparent, comprehensive, and unbiased evidence base [31]. Unfortunately, the present review of field studies conducted in Mexico showed potential publication bias. To the best of our knowledge, in the last ten years, 13 systematic reviews of malocclusion prevalence in healthy populations have been published: three studies on the worldwide prevalence of malocclusion [29,30,32], one study on Iranian children [33], one on Iranian population [34], one on Turkish children and adolescents [35], two on Chinese schoolchildren [36,37], two on Indian children and adolescents [38,39], one on Kingdom of Saudi Arabia [40], one on Nation-wide Saudi Arabia [41], and one on the Indigenous population in Brazil [42]. Of them, only one review performed the bias publication assessment [37].

Publication bias in malocclusion prevalence studies conducted in Mexico may stem from several structural and academic factors, including limited funding for epidemiological research, a high proportion of undergraduate or postgraduate theses that remain unpublished, and preferential publication of studies reporting higher or more clinically striking prevalence estimates. In addition, variability in study design, non-standardized diagnostic criteria, and selective reporting of outcomes may further contribute to the biased dissemination of results. These challenges are not unique to Mexico and are similarly observed in other low- and middle-income settings, where the gray literature constitutes a substantial proportion of the available evidence [43]; however, they appear to be less prominent in regions with a stronger research infrastructure and routine registration of observational studies. These highlight the need for more comprehensive training of clinical dental professionals in research methodologies. Such training should include study design, assessment of risk of bias, and principles of high quality, complete, transparent, and rigorous reporting. Strengthening these competencies would contribute to the generation of more reliable evidence and enhance the scientific value and credibility of research findings in dentistry.

The overall low certainty of the evidence observed in this review mainly arises from methodological limitations that are commonly found in epidemiological studies of malocclusion. Using a GRADE approach, the overall certainty of the prevalence estimate was rated low. The main limitations were the high proportion of included studies in the meta-analysis with a high risk of bias (44%) and the very high statistical heterogeneity (I^2^ = 97.9%), which could not be explained by subgroup analyses. Additionally, the 95% confidence interval was wide (39–61%), indicating imprecision. Also, there is suggestive publication bias LFK > 1. Therefore, the true prevalence of malocclusion may be substantially different from the pooled estimate. Using the Hoy et al. tool, the main issue was observed in the external validity item and it contributed substantially to the risk of bias. Many malocclusion prevalence studies rely on convenience samples, such as school-based or clinic-based populations, which limits representativeness and compromises external validity. Another important limitation affecting external validity is the lack of random selection procedures in the included studies.

Several statistical methods for detecting publication bias are available. Standard approaches include funnel plots, Egger’s test, Begg’s test, skewness analysis, and the trim-and-fill method. However, publication bias assessment is seldom performed, primarily because many dentistry-related meta-analyses include a limited number of studies and exhibit substantial clinical and methodological heterogeneity. These characteristics reduce the validity of conventional funnel plot and *p*-value-based approaches. In the context of the present review, the LFK index and the Doi plot represent more appropriate and robust methods. The LFK approach has been shown to outperform conventional methods in detecting asymmetry in meta-analyses of proportions and provides an objective interpretation using predefined thresholds [44]. In that sense, integrating publication bias assessment would strengthen the transparency, robustness, and applicability of evidence synthesis in dental public health research.

On the other hand, in Mexico, malocclusion has not yet been incorporated into public oral health policies, and orthodontic care is largely delivered through the private sector. One potential challenge in recognizing malocclusion as a public oral health concern is the limited availability of systematically organized evidence and comprehensive epidemiological analyses. Consequently, the relatively scarce number of rigorously conducted and transparently reported studies on malocclusion prevalence may limit the extent to which prevention, early detection, and timely management of malocclusions are considered within health policy frameworks. Over time, this situation could be associated with the persistence of oral health inequalities [45].

Future research on malocclusion prevalence would benefit from improved methodological standardization. The use of uniform and validated diagnostic criteria, stage of dentition, and representative population-based sampling strategies would enhance comparability across studies and reduce heterogeneity. In addition, national level epidemiological surveys conducted under standardized protocols would provide more reliable estimates to inform clinical practice and public oral health policies.

### Strengths and Limitations

This study has several strengths. It represents, to our knowledge, the first systematic reviews to specifically evaluate publication bias in epidemiological studies of malocclusion in a Mexican population. The use of a comprehensive search strategy, independent data extraction, and a validated risk of bias tool for prevalence studies strengthens the methodological rigor of the review. In addition, the application of a random-effects model and the use of the LFK index and Doi plot allowed for a more appropriate assessment of heterogeneity and publication bias in the prevalence meta-analysis.

However, some limitations should be acknowledged. One limitation of this meta-analysis relates to the homogenization of outcomes for analysis. Our approach, which used the Angle classification and the DAI, involved dichotomization and may oversimplify the clinical reality of Class I malocclusion, as some individuals with Class I relationships still present meaningful dental irregularities that require orthodontic treatment. Nevertheless, this decision was necessary to enable quantitative synthesis across studies. In addition, most of the included studies exhibited a moderate to high risk of bias, imprecision, and publication bias, which contributed to the low certainty of the evidence. Furthermore, substantial methodological heterogeneity and limited data availability may have influenced the pooled estimates.

## 5. Conclusions

In Mexico, there is a high prevalence of malocclusions among children and adolescents. However, the included studies showed low certainty of the evidence, due to the risk of bias and the potential presence of publication bias. Therefore, the available findings should be interpreted with caution and are not sufficiently robust to support definitive conclusions. Consequently, there is a critical need to improve the quality of observational prevalence studies. Publication bias misleads the available evidence for the prevalence of malocclusions, leading to erroneous conclusions about the magnitude of associations in epidemiological research and thereby affecting public health decisions. Consequently, identifying and addressing publication bias is critical to ensuring that systematic reviews and meta-analyses yield valid, unbiased conclusions.

## Figures and Tables

**Table 1:** Eligibility criteria according to PICO strategy and keywords used for the search strategy.

PICO Strategy	
Population	Mexican children ≤ 15 years old
Outcome	Prevalence rates of malocclusions
Study design	Observational studies (prospective and retrospective studies)
Electronic database	MEDLINE/PubMed, Dentistry & Oral Sciences Source, Scopus, Google Scholar, and Web of Science
Focused question	What is the publication bias of evidence about the prevalence of malocclusion in Mexican children?
Algorithms and keywords used	
PubMed	(malocclusion OR malocclusions) AND (prevalence) AND (Mexico)
Dentistry & Oral Sciences Source	(malocclusion OR malocclusions) AND (prevalence) AND (Mexico)
Google Scholar	(malocclusion OR malocclusions) AND (prevalence) AND (Mexico)
Web of Science	#1TS=(malocclusion OR malocclusions) #2TS=(prevalence) #3TS=(Mexico)
Scopus	TITLE-ABS-KEY ((malocclusion OR malocclusions) AND (prevalence) AND (Mexico))

**Table 2:** Quality of evidence according to GRADE.

Certainty Assessment						
Malocclusions in Mixed Dentition						
№ of Participants (Studies)	Risk of Bias	Inconsistency	Indirectness	Imprecision	Publication Bias	Overall Certainty of Evidence
18	Very serious ^a^	Very serious ^b^	Not serious	Serious ^c^	Publication bias suspected	⨁⨁◯◯ Low

Explanations: ^a^ Moderate–high risk of bias due to external validity; ^b^ due to the high heterogeneity; and ^c^ wide intervals. The symbol “⨁” represents a point indicating confidence in the effect estimate, while the hollow circle “◯” indicates a point where that confidence is lacking. Therefore, ⨁⨁○○ (two plus signs followed by two circles) is the internationally recognized symbol for Low quality of evidence.

## Data Availability

No new data were created or analyzed in this study. Data sharing is not applicable to this article.

## Appendix A

**Table A1:** Excluded studies with reasons.

9	Reason
(1) Flores-Longoria, 2002 [46] Thesis	Participants were not stratified by age
(2) Beraud-Osorio et al., 2004 [47] Article	Diagnosis of posterior crossbite
(3) Casanova-Rosado et al., 2005 [48] Article	Participants were not stratified by age
(4) Gutierrez-Juárez and Gutiérrez-Venegas, 2006 [49] Article	Participants were not stratified by age
(5) Álvarez-Neira et al., 2006 [50] Article	Severity analysis in previously diagnosed patients.
(6) Ramírez-Velásquez et al., 2007 [51] Article	Participants were not stratified by age
(7) Pinzón-Te, 2007 [52] Thesis	Participants were not stratified by age
(8) Najera-Avila, 2008 [53] Thesis	Participants were not stratified by age
(9) López-Pérez et al., 2008 [54] Article	Diagnosis of anterior open bite
(10) Marroquín-Rodríguez et al., 2010 [55] Article	Participants outside the eligible age range
(11) Murrieta-Pruneda et al., 2011 [56] Article	Participants outside the eligible age range
(12) Taboada-Aranza et al., 2011 [57] Article	Participants were not stratified by age
(13) Ramírez-Mendoza et al., 2012 [58] Article	Participants were not stratified by age
(14) Padilla-Díaz et al., 2013 [59] Article	Participants were not stratified by age
(15) De la Peña-Méndez et al., 2013 [60] Thesis	Participants were not stratified by age
(16) Guerrero-Castellón and Aguilar-Fuentes, 2013 [61] Article	Participants were not stratified by age
(17) Mendoza-Oropeza et al., 2014 [62] Article	Participants were not stratified by age
(18) Reyes-Ramírez et al., 2014 [63] Article	Participants were not stratified by age
(19) Rueda-Ventura and Isidro-Olán, 2014 [64] Article	Participants were not stratified by age
(20) Moreno-Rojas et al., 2015 [65] Article	Participants were not stratified by age
(21) Aamodt et al., 2015 [66] Article	Participants were not stratified by age
(22) Favela-Campa, 2015 [67] Thesis	Participants were not stratified by age
(23) Lozano-Longoria, 2015 [68] Thesis	Participants were not stratified by age
(24) Garza-Ramos, 2016 [69] Thesis	Without prevalence information
(25) Aguila-García and Ortíz-Bravo, 2016 [70] Thesis	Participants were not stratified by age
(26) Castillo-Carmona et al., 2016 [71] Article	Participants were not stratified by age
(27) Gutiérrez-Rojo et al., 2016 [72] Article	Participants were not stratified by age
(28) Orozco-Cuanalo et al., 2016 [73] Article	Participants were not stratified by age
(29) García-Garza et al., 2017 [74] Article	Patients with cleft lip and palate
(30) Ramos-Ríos et al., 2017 [75] Article	Diagnosis of anterior open bite in asthmatic patients
(31) Torres-Capetillo et al., 2018 [76] Article	Participants were not stratified by age
(32) Rodriguez-Morales, 2018 [77] Thesis	Participants were not stratified by age
(33) Garibo-Ruíz et al., 2018 [78] Thesis	Participants were not stratified by age
(34) Martínez-Ortíz et al., 2019 [79] Article	Participants were not stratified by age
(35) Chapa-Hernández, 2019 [80] Thesis	Diagnosis of opening limitations
(36) Silva and Kang, 2001 [81] Article	Participants living in California
(37) Murrieta-Pruneda et al., 2012 [82] Article	Participants outside the eligible age range
(38) Gutiérrez-Rojo et al., 2015 [83] Article	Participants outside the eligible age range
(39) González-Amaral and Rodríguez-López, 2018 [84] Article	Participants outside the eligible age range
(40) Tiburcio-Morteo et al., 2021 [85] Article	Participants outside the eligible age range
(41) Tristán-López, 2019 [86] Thesis	Severity analysis in previously diagnosed patients
(42) Marín-Delgado et al., 2019 [87] Article	Incomplete data

**Table A2:** Characteristics of the individual studies and their results (mixed dentition).

ID	Population	Result
**Roa-Gonzalez, 2002 [88]** **Thesis**	Dental medical records of patients aged 7–12 years (n = 281), Monterrey, Nuevo León state. Age (mean): 8.76 ± 1.33 years Male: 48.4% (n = 136) Female: 51.6% (n = 145)	**Molar relationship** (Angle classification) Class I: 43.8% (n = 123) Class II 20.6% (n = 58) Class III: 35.6% (n = 100)
**Montiel-Jaime, 2004 [89]** **Article**	Children aged 6–12 years (n = 135), Netzahualcoyotl city. N/A	**Molar relationship** (Angle classification) Class I: 68% (n = 92) Class II: 23.88% (n = 31) Class III: 9% (n = 12) **Bite abnormalities** Anterior open bite: N/A Posterior open bite: N/A Anterior crossbite: N/A Posterior crossbite: N/A Edge-to-edge bite: N/A Overjet: N/A Overbite: N/A
**Orozco-Cuanalo et al., 2004 [90]** **Article**	Schoolchildren aged 6–13 years (n = 888), Netzahualcoyotl city. Age (mean): 8.5 years Female: 47.2% Male: 52.8%	**Molar relationship** (Angle classification) Class I: N/A Class II: N/A Class III: N/A **Bite abnormalities** Total: 75.90% (n = 674). Anterior crossbite: 15.9% Posterior crossbite: 14.3% Deviated dental midline: 35.7% Edge-to-edge bite: 6.3% Tooth crowding: 36.5% Ectopic eruption: 17.5% Gyroversion: 27.8% Anterior open bite and diastema: 8.7%
**Talley-Millán et al., 2007 [22]** **Article**	Patients aged 8–40 years (n = 428), Postgraduate dental school clinic (DEPeI), National Autonomous University of Mexico (UNAM), Mexico City. Age (mean): 16.86 years	**Molar relationship** (Angle classification) Class I: 52.8% (n = 226) 8–12 years old: 12.9% (n = 55) * 13–19 years old: 27.8% (n = 119) 20–40 years old: 12.1% (n = 52) Class II: 33.9% (n = 145) 8–12 years old: 6.1% (n = 26) * 13–19 years old: 8% (n = 77) 20–40 years old: 9.8% (n = 42) Class III: 13.3% (n = 57) 8–12 years old: 1.6% (n = 7) * 13–19 years old: 7% (n = 30) 20–40 years old 20 (4.7%)
**Aguilar Díaz et al., 2011 [91]** **Article**	Male children aged 8–10 years (n = 212) San Luis Potosí state.	**Molar relationship** (DAI) No anomalies: 30.7% (n = 65) Total abnormalities: 69.3% (n = 147) [Definite malocclusion: 34.4% (n = 73), Severe Malocclusion: 16.5% (n = 35), and Very Severe: 18.4% (n = 39)].
**Mares-Favela, 2012 [92]** **Thesis**	Male children aged 6–13 years (n = 425) Monterrey, Nuevo León state. Male: 56.86% (n = 224) Female: 47.29% (n = 201)	**Molar relationship** (Angle classification) Class I: 62.12% (n = 264) Class II: 31.06% (n = 132) Class III: 6.82% (n = 29)
**Ramírez-Mendoza et al., 2012 [93]** **Article**	Students from two primary schools aged 6–12 years (n = 273), Villahermosa, Tabasco state.	**Molar relationship** (Angle classification) No alterations 25% (n = 67) Alterations: 75% (n = 206)
**Sierra-Saavedra, 2012 [94]** **Thesis**	Male primary school students aged 6–12 years (n = 498), San Nicolás de los Garza, Nuevo León state.	**Molar relationship** (Angle classification) Class I: (n = 368) Class II: (n = 93) Class III: (n = 37)
**Aguilar-Moreno and Taboada-Aranza, 2013 [95]** **Article**	Students from elementary schools (n = 375), Nezahualcóyotl and Izcalli cities. Age (mean): 8.8 (±1.7) Male: 50.1% (n = 188) Female: 49.9% (n = 187)	**Molar relationship** (Angle classification) Class I: 55.2% (n = 207) Class II div.1: 19.2% (n = 72) Class II div. 2: 15.7% (n = 59) Class III: 9.9% (n = 37)
**Silva-Flores et al., 2013 [96]** **Article**	Schoolchildren aged 7–12 years (n = 402), Tamaulipas. Age (mean): 9.5 years ±1.5 Female: (n = 208) Male: (n = 194)	**Molar relationship** N/A **Bite abnormalities:** Horizontal overbite: (n = 27) Vertical overbite: (n = 25) Posterior crossbite: (n = 15) Anterior crossbite: (n = 23) Open anterior bite: (n = 19) Edge-to-edge bite: (n = 18)
**Sánchez-Pérez et al., 2013 [97]** **Article**	Schoolchildren aged 15 years (n = 249), Mexico City. Male (n = 131) Female (n = 118)	**Molar relationship** (DAI index) No anomalies: 67.9% (n = 169) Anomalies: 32.1% (n = 80) [Definite malocclusion: 18.5% (n = 46), Severe Malocclusion: 7.6% (n = 19) and Very Severe: 6.0% (n = 15)]. **Bite abnormalities** Overjet: 61% Open bite: 4.5%
**Estrada-González, 2013 [98]** **Thesis**	Schoolchildren aged 6–15 years (n = 400), Monterrey, Nuevo León state. Male: 42.5% (n = 170) Female: 57.5% (n = 230)	**Molar relationship** (Angle classification) Class I: 66.25% (n = 265) Class II: 24.62% (n = 98) Class III: 9.13% (n = 37)
**Sarellano-Castillo, 2013 [99]** **Thesis**	Schoolchildren aged 8–11 years (n = 382), Nuevo León state. 9 years old: 35% 8–10 years old: 28% 7–11 years old: 5%	**Molar relationship** (Angle classification) Class I: 55% (n = 212) Class II: 36% (n = 139) Class III: 8% (n = 31)
**Silva-Pérez et al., 2014 [100]** **Article**	Schoolchildren aged 6–12 years (n = 680), Tabasco. Female: (n = 386) Male: (n = 359)	**Molar relationship** (Angle classification) Normal occlusion: 30.3%(n = 206) Alterations: 69.7% (n = 474)
**Padilla-Corona, 2014 [101]** **Thesis**	Schoolchildren aged 6–12 years (n = 749), Tamaulipas state. Age (mean): 8.44 ± 1.77 years N/A	**Molar relationship** (Angle classification) Class I: 63.41% (n = 475) Class II: 26.81% (n = 201) Class III: 9.74% (n = 73)
**Tokunaga-Castañeda et al., 2014 [23]** **Article**	Dental medical records of patients aged 8–40 years (n = 428), DEPeI, UNAM. Age (mean): 16.85 years Female: 64.7% (n = 277) Male: 35.3% (n = 151) 8–12 years old: 20.6% (n = 88) * 13–19 years old: 52.8% (n = 226) 20–40 years old: 26.6% (n = 114)	**Molar relationship** (Angle classification) Class I: 8–12 years old: 8.9% (n = 38) * 13–19 years old: 29% (n = 124) 20–40 years old: 15.4% (n = 66) Class II: 8–12 years old: 9.3% (n = 40) * 13–19 years old: 18.2% (n = 78) 20–40 years old: 9.6% (n = 41) Class III: 8–12 years old: 2.3% (n = 10) * 13–19 years old: 5.6% (n = 24) 20–40 years old: 1.6% (n = 7)
**Alcaraz-Gutiérrez, 2016 [102]** **Thesis**	Male children aged 9–11 years (n = 543), Tepatitlán de Morelos, Jalisco state. Age (mean): 9.8 years Male: 40.5% (n = 222) Female: 59.5% (n = 323)	**Bite abnormalities** (Modified WHO index) None: 41.4% (n = 225) Small: 56.7% (n = 308) Moderate or Severe: 1.8% (n = 10)
**Gálvez-Armenta and Velásquez-Luna, 2016 [103]** **Article**	Dental medical records of patients aged 6–12 years (n = 179), Sinaloa state. Age (mean): 7.9 ± 1.4 Female: (n = 95) Male: (n = 84)	**Molar relationship** (Angle classification) Class I: 79% (n = 92) Class II: 17% (n = 20) Class III: 4% (n = 5) **Bite abnormalities** Edge-to-edge bite: 11% Deep overbite: 10% Overjet: 6% Anterior open bite: 5% Anterior crossbite: 4% Posterior crossbite: 2%
**Castrejón-Díaz, 2017 [104]** **Thesis**	Students aged 12–15 years (n = 400), Culiacan, Sinaloa state. Age (mean): 8.90 ± 1.39 Male: 46.2% (n = 185) Female: 53.8% (n = 215)	**Molar relationship** (DAI index) No anomalies: 22.5% (n = 90) Anomalies: 77.5% (n = 310) [Definite malocclusion: 20% (n = 80), 12 years old: 21.5% (n = 43), 15 years old: 18.5% (n = 37) Severe malocclusion: 24.3% (n = 97), and Very Severe: 33.2% (n = 133)].
**Chul-Back et al., 2018 [105]** **Article**	Male children aged 8–14 years (n = 526), Montemorelos, Nuevo León state. Female: (n = 247) Male: (n = 279)	**Molar relationship** ND **Bite abnormalities** Total: 54.4% (n = 286) Overjet: 24% (n = 126) Edge-to-edge bite: 18.1% (n = 95) Anterior crossbite: 3.6% (n = 19) Anterior normal occlusion: 54.4% (n = 286) Overjet: 24% (n = 126) *p* < 0.05 Edge-to-edge bite: 18.1% (n = 95) Anterior crossbite: 3.6% (n = 19)
**Rodríguez-Cardoso, 2018 [77]** **Thesis**	Male children aged 6–12 years (n = 93), Puebla city. Male: 61.3% (n = 57) Female: 38.7% (n = 36)	**Molar relationship** (Angle classification) Class I: 21.5% (n = 20) Class II: 54.83% (n = 51) Class III: 23.65% (n = 22)
**González-Aragón et al., 2020 [106]** **Article**	Teenagers aged 13–15 years (n = 424), Mexico City. Age (mean): 13.7(±0.44) Female: (n = 225) Male: (n = 199)	**Molar relationship** N/A **Bite abnormalities** Open bite: 1.7% (n = 7) Overbite: 11.6% (n = 42) Posterior crossbite: 7.6% (n = 32) Anterior crossbite: 7.8% (n = 33) Overjet: 18.9% (n = 80)
**García-Pérez et al., 2021 [107]** **Article**	Male child patients aged 8–10 years (n = 480), Dental clinic of School of Higher Studies (FES) Iztacala, UNAM, Tlalnepantla de Baz, State of Mexico. Age (mean): 9.2(±0.75) Male: 50.6% (n = 243) Female: 49.4% (n = 237)	**Molar relationship** (DAI index) No anomalies: 20.6% (n = 99) Anomalies: 79.4% (n = 381) [Definite malocclusion: 31.3% (n = 150), Severe Malocclusion: 25.6% (n = 123), and Very Severe: 22.5% (n = 108)]. **Bite abnormalities** Anterior open bite: 4.4% (n = 21) Anterior crossbite: 45.6% (n = 219)

* Data included in the meta-analysis. No data available or incomplete data: (N/A).

**Table A3:** Characteristics of individual studies and their results (Deciduous dentition).

ID	Population	Result
**Medrano-Luna et al., 2002 [108]** **Article**	Children aged 3–5 years (n = 193), Iztapalapa, Mexico City.	**Terminal plane** Flush terminal plane and mesial step: 81.9% (n = 158) Distal step and mesial step with tendency to increase: 18.1% (n = 35) **Bite abnormalities** ND
**Vásquez-Nava et al., 2006 [109]** **Article**	Male children aged 4–5 years (n = 1160), Tamaulipas state. Male: 50.2% Female: 49.8%	**Terminal plane** ND **Bite abnormalities** Total: 55.2% (n = 640) Anterior open bite: 51.03% Posterior crossbite: 7.5%
**Hernández-Chacón et al., 2014 [26]** **Article**	Children aged 3–5 years (n = 144), Juárez City, Chihuahua state. Female: 47% (n = 68) Male: 53% (n = 76)	**Terminal plane** Mesial step: 56% (n = 81) Flush terminal plane and mesial step with tendency to increase: 19% (n = 27) Distal step: 6% (n = 9) **Bite abnormalities** Total: 95.1% (n = 137) Overbite: 54.16% (n = 78) Crossbite: 27.08% (n = 39) Open bite: 9.72% (n = 14) Edge-to-edge bite: 4.16% (n = 6)
**Manzo-Palomera et al., 2018 [110]** **Article**	Preschooler aged 3–5 years (n = 522), North Coast Region, Jalisco state. Female: 49.24% Male: 50.76%	**Terminal plane** N/A **Bite abnormalities** No malocclusion: 69.16% (n = 361) Mild to severe malocclusion: 30.84% (n = 161) [Mild degree malocclusions: 26.05% (n = 136) and Moderate to severe degree malocclusion: 4.79% (n = 25)].
**Aldaz-Medina, 2018 [25]** **Thesis**	Children patients aged 3–5 years (n = 100), Postgraduate dental clinic of Medicine School of the Autonomous University of Querétaro (FMUAQ), Queretaro city. Male: 45% Female: 55%	**Terminal plane** N/A **Bite abnormalities** Open bite: (n = 8) Posterior crossbite: (n = 5) Anterior crossbite: (n = 14)
**Rodríguez-Hernández, 2020 [111]** **Thesis**	Children aged 1–3 years (n = 95), Autonomous University of Baja California (UABC), Campus Tijuana, Tijuana city. Female: 48.4% (n = 46) Male: 51.5% (n = 49)	**Terminal plane** N/A **Bite abnormalities** Total: 24.2% (n = 23) Anterior open bite: 8.4% (n = 8) Crossbite posterior: 2.1% (n = 2) Horizontal overbite: 13.7% (n = 13)
